# Ultrasound-based bone age assessment in children and adolescents: a mini-review

**DOI:** 10.3389/fped.2025.1615923

**Published:** 2025-08-21

**Authors:** Yixuan Zhang, Xiao Yang, Li Zhang, Zeqing Zhao, Yamei Yang

**Affiliations:** Department of Ultrasound, Peking Union Medical College Hospital, Chinese Academy of Medical Sciences, Beijing, China

**Keywords:** bone age assessment, ultrasound imaging, skeletal maturity, children and adolescents, pediatric growth evaluation

## Abstract

Bone age assessment is a critical tool for evaluating skeletal maturity in children and adolescents, with implications for growth monitoring and clinical decision-making. While traditional radiographic methods such as the Greulich-Pyle and Tanner-Whitehouse systems remain the gold standard, concerns over ionizing radiation exposure have spurred interest in ultrasound-based alternatives. This mini-review synthesizes current evidence on ultrasound bone age assessment, highlighting its advantages as a radiation-free, non-invasive modality with strong correlations to radiographic standards. Key advancements include standardized scoring systems, ossification ratios (e.g., radius/ulna/femur), and acoustic measures (e.g., speed of sound), which enhance reliability and reduce inter-operator variability. However, challenges persist, including protocol standardization, population-specific variability, and operator dependency, particularly in advanced pubertal stages. Future directions emphasize the development of large-scale, longitudinal and multi-ethnic reference databases, consensus guidelines, and AI integration to improve precision. Ultrasound bone age assessment shows promise as a viable clinical tool, but further refinements are needed to address its limitations and ensure equitable applicability across diverse populations.

## 1 Introduction

Bone age assessment is essential for evaluating the skeletal maturity of children and adolescents, providing insights into their growth potential. Unlike chronological age, bone age reflects biological maturity and is determined by examining bone ossification, particularly in secondary ossification centers, which develop with age in the ends of long bones. This evaluation helps predict an individual's final adult height; delayed bone age suggests more growth potential compared to advanced bone age, provided other factors such as nutrition and hormones are normal (1). Moreover, bone age assessment plays a vital role in monitoring pubertal progression. Puberty is a period of rapid growth and development, and skeletal maturation is closely linked to hormonal changes during this time. Assessing bone age can provide valuable information about the timing and pace of puberty, which has implications for physical development (2).

Traditional methods for bone age assessment primarily rely on radiographic imaging (3). Two of the most widely used methods are the Greulich-Pyle (GP) and Tanner-Whitehouse (TW) methods (4). The GP method involves comparing a patient's hand-wrist radiograph to a standardized atlas of reference images that represent specific bone ages from infancy to skeletal maturity. The clinician matches the patient's radiograph to the closest reference image to assess skeletal development. The TW method is a quantitative approach that scores the maturity of bones based on specific criteria. It assigns numerical scores for different ossification stages, which are summed and converted to bone age using standardized tables. Traditional radiographic methods for bone age assessment have notable limitations, particularly concerning radiation exposure in pediatric populations. While a single hand-wrist x-ray involves low radiation, repeated assessments can lead to cumulative exposure, increasing the risk of radiation-related health issues (5). This is particularly concerning for children and adolescents, who are more sensitive to the effects of ionizing radiation than adults.

Given the limitations of traditional radiographic methods, there is a growing interest in developing alternative approaches for bone age assessment. Ultrasound (US) has emerged as a promising non-ionizing imaging technique for evaluating skeletal maturity in adolescents (6). One of the primary advantages of US is that it does not involve ionizing radiation, making it a safe alternative to radiography, especially for pediatric populations (5). This eliminates the risk of cumulative radiation exposure associated with repeated radiographic assessments.

This article will first outline the fundamental principles of ultrasound bone age assessment, followed by a comparison between ultrasound and traditional radiological methods. It will then address current challenges and future development directions, ultimately leading to a conclusion.

## 2 Methods

This narrative mini-review synthesizes 20 researches on ultrasound-based bone age assessment in children and adolescents, identified through PubMed searches with keywords such as “bone age,” “skeletal maturity,” and “ultrasound.” The review emphasizes English-language studies evaluating ultrasound techniques, encompassing sample sizes ranging from 24 to 1,089 participants. As a narrative review, it forgoes formal systematic methods, enabling a flexible synthesis of studies to showcase both technological advancements and ongoing challenges in the field. Additionally, we briefly mentions the included researches' limitations, such as small sample sizes, operator bias, or the absence of blinding.

## 3 Fundamentals of ultrasound-based bone age assessment

### 3.1 Basic physical principles of ultrasound imaging in bone age assessment

Ultrasound imaging is a non-invasive technique that uses high-frequency sound waves to create images of internal structures. It works by transmitting sound waves into the body and receiving echoes reflected by tissues, which vary based on properties such as density and elasticity. Echogenicity indicates a tissue's ability to reflect ultrasound: high echogenicity (e.g., bone) appears bright, while low echogenicity (e.g., soft tissues) appears darker. Attenuation, caused by absorption and scattering, reduces the beam's intensity and can affect image quality, especially with dense tissues such as bone. In bone age assessment, ultrasound focuses on ossification centers and epiphyseal regions, where the strong reflections at bone-soft tissue boundaries enhance imaging of bone surfaces (7).

Epiphyseal regions and ossification centers are critical anatomical landmarks for determining skeletal maturity in adolescents (1). The epiphyseal region, located at the ends of long bones, is initially composed of cartilage and gradually ossifies over time. Ossification centers are the areas within the epiphysis where bone formation begins. Ultrasound offers a non-ionizing alternative for assessing skeletal maturity, which can effectively visualize the epiphyseal cartilage and ossification centers due to the difference in acoustic impedance between cartilage, bone, and surrounding soft tissues. The configuration of ossification centers changes with growth and maturity. Initially, ossification centers appear as small, distinct regions within the epiphysis. As the adolescent matures, these centers enlarge and eventually fuse with the metaphysis, indicating the completion of skeletal growth. Ultrasound allows for real-time visualization of these changes, providing valuable information for bone age assessment (8, 9). Compared to radiographic methods, ultrasound offers the advantage of dynamic imaging, allowing the examiner to assess the stability and movement of the joint, as well as the surrounding soft tissues.

### 3.2 Key ultrasound parameters and quantitative measurements

#### 3.2.1 Ultrasound scoring system for bone age assessment

Ultrasound-based scoring systems for bone age assessment provide an effective method in translating raw imaging data into standardized metrics that align with clinical decision-making (10).

Schmidt et al. (11) firstly conducted a comprehensive study on the ossification stages of the distal radius in 615 participants aged 10–25 years. The findings showed that ultrasound could provide meaningful information for forensic age estimation, with high interobserver agreement (weighted kappa = 0.898). In a study by Ağırman et al. (9), the scoring system was used assess bone age and pubertal growth in 120 children aged 10–17 years, which divided the images into 5 levels based on the ultrasound findings of the epiphysis. The ultrasound images were acquired before hand-wrist radiographs were evaluated. The results showed strong correlations between ultrasound and radiographic bone age assessments. Ekizoglu et al. (5) further explored the use of ultrasound stages for forensic age estimation in a larger sample of 688 individuals aged 9–25 years. The study found that ultrasound could accurately determine critical legal age limits of 14 and 15 years, with high interobserver reliability (Cohen's kappa = 0.919). These results suggested that ultrasound is a feasible method for bone age assessment.

#### 3.2.2 Ossification ratio (ossification center/epiphysis dimensions)

Ossification ratio is a quantitative measurement used in ultrasound-based bone age assessments to evaluate skeletal maturity. The ratio is determined by measuring the dimensions of both the ossification center and the epiphysis in specific bones, such as the radius, ulna, and femur (12). Specifically, the ossification ratio is defined as the height of the epiphyseal ossification center divided by the total height of the epiphysis, including its cartilaginous component. The methodology for measuring ossification ratios involves acquiring ultrasound images of the selected skeletal sites (13). As a child grows and matures, the ossification center enlarges, resulting in an increased ossification ratio. Therefore, a higher ossification ratio indicates a more advanced stage of skeletal maturity.

Several studies have highlighted the predictive validity of ossification ratios in determining bone age. For example, ossification ratios were firstly measured using B-mode ultrasound (12). The same research group further developed Skeletal Maturity Scores (SMS) (i.e., the summation of ossification ratios of the radius, ulna, and femur multiplied by 100) to evaluate bone age, achieving impressive sensitivity and specificity when identifying abnormal bone age relative to radiographic standards (13). Furthermore, the SMS system exhibited exceptional performance in predicting final adult height attainment, evidenced by area under the curve (AUC) values of 0.99 for boys and 0.95 for girls (*n* = 120) (14). Notably, the findings revealed that some children ceased height growth before achieving full ossification.

#### 3.2.3 Quantitative ultrasound (QUS): speed of sound (SOS) and broadband ultrasound attenuation (BUA)

Speed of Sound (SOS) and Broadband Ultrasound Attenuation (BUA) are two key acoustic measures used in quantitative ultrasound (QUS) to evaluate bone density and quality (15). These parameters provide information about the microarchitecture and composition of bone, which are important determinants of bone strength and fracture risk.

SOS refers to the velocity at which ultrasound waves travel through bone. It is influenced by bone density, elasticity, and microstructure (16). Higher SOS values generally indicate denser and more rigid bone (17). BUA measures the rate at which ultrasound waves lose intensity as they propagate through bone. Attenuation is primarily caused by absorption and scattering of ultrasound waves by the bone matrix. Higher BUA values indicate greater attenuation, which can be associated with increased bone porosity or altered bone microstructure.

Studies have shown that SOS in the radius and tibia is closely linked to chronological age (16). Notably, a weak but statistically significant correlation between skeletal age and SOS was found (15). Leveraging SOS technology, the BonAge system has been developed to provide assessments of bone age and applied to clinical environment (17). A research in 1,001 Colombian children and adolescents aged 9–17.9 years has established gender- and age-specific reference values for broadband ultrasound attenuation (BUA), demonstrating its utility in monitoring bone development (18). Interestingly, peak BUA values have been identified at 19 years in males and 16 years in females, highlighting important differences in bone maturation across genders (19).

Research has demonstrated a strong association between SOS in the radius and tibia and chronological age (16). Additionally, a weak yet statistically significant correlation between skeletal age and SOS has been observed (15). Leveraging SOS technology, the BonAge system has been developed, providing assessments of bone age that can be effectively applied in clinical settings (17).

A study conducted in Colombia involving children and adolescents aged 9–17.9 years has established gender- and age-specific reference values for BUA. This highlights the potential of BUA as a valuable tool for monitoring bone development (18). Notably, peak BUA values were found to occur at 19 years in males and at 16 years in females, underscoring significant differences in bone maturation between genders (19).

In summary, these findings emphasize QUS's implications for clinical practice and monitoring of skeletal development.

#### 3.2.4 Ultrasound bone age determination adapted from radiographic methodology

The traditional reliance on radiographic methods, particularly the GP atlas and the TW system, continues to underpin bone age assessment (3). These established approaches, based on criteria such as ossification and epiphyseal fusion, have served as a foundation for decades. Recently, advancements in the field have focused on adapting and refining these methods for ultrasound-based applications. For example, one study demonstrated the feasibility of applying an ultrasound-based adaptation of the GP atlas for bone age assessment (8). Similarly, new conversion formulas have been developed to accurately derive TW skeletal maturity scores directly from ultrasound images (20). Building on these innovations, Cumming et al. introduced the BAUSport system, which leverages ultrasound data processed through an algorithm grounded in the TW2 scoring method to estimate skeletal age (21). These developments underscore a significant shift in the landscape of bone age assessment, where traditional radiographic techniques are being reimagined and optimized for non-radiographic modalities, offering safer and more adaptable alternatives.

**Figure 1:**
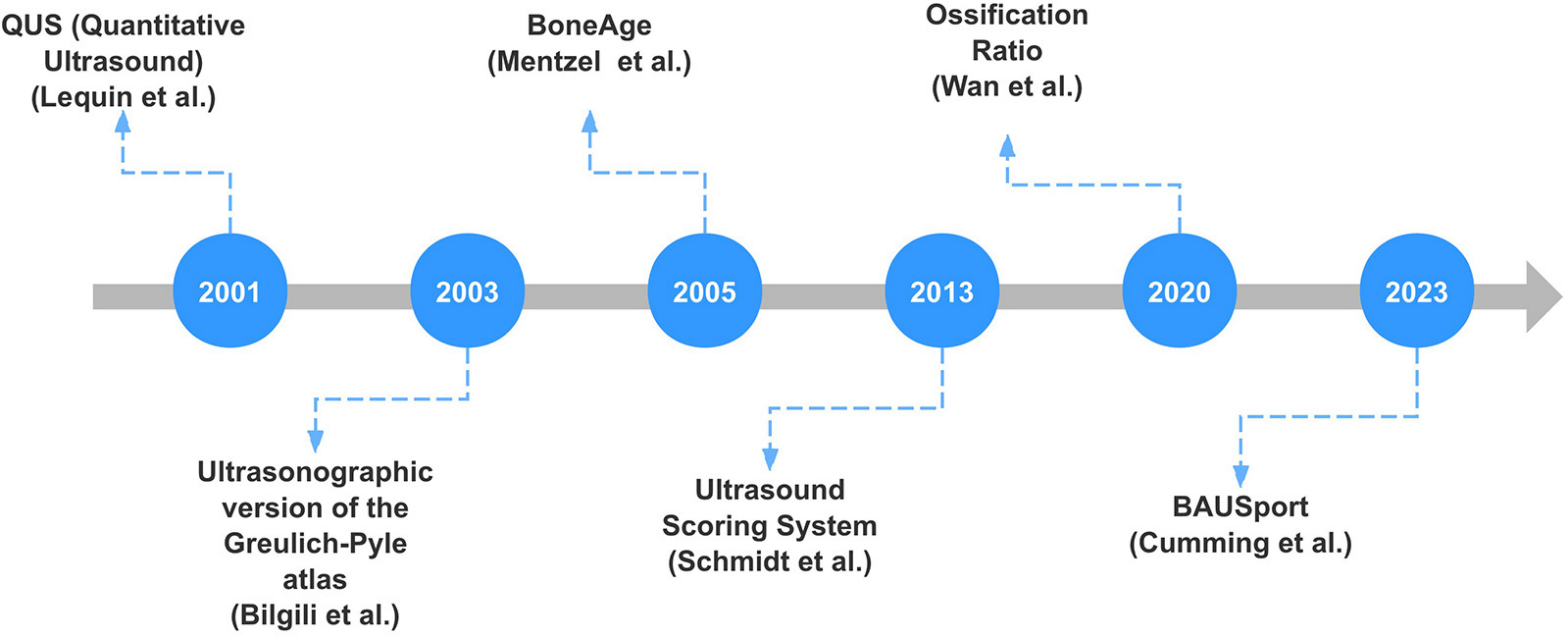
Timeline of significant advancements in ultrasound-based bone age assessment.

Figure 1 shows the timeline of significant advancements in ultrasound bone age assessment over recent years. Each milestone represents a notable contribution to the field, highlighting the progression and innovation in ultrasound techniques for bone age assessment.

### 3.3 Commonly scanned skeletal sites and multi-site assessments for reliability

**Figure 2:**
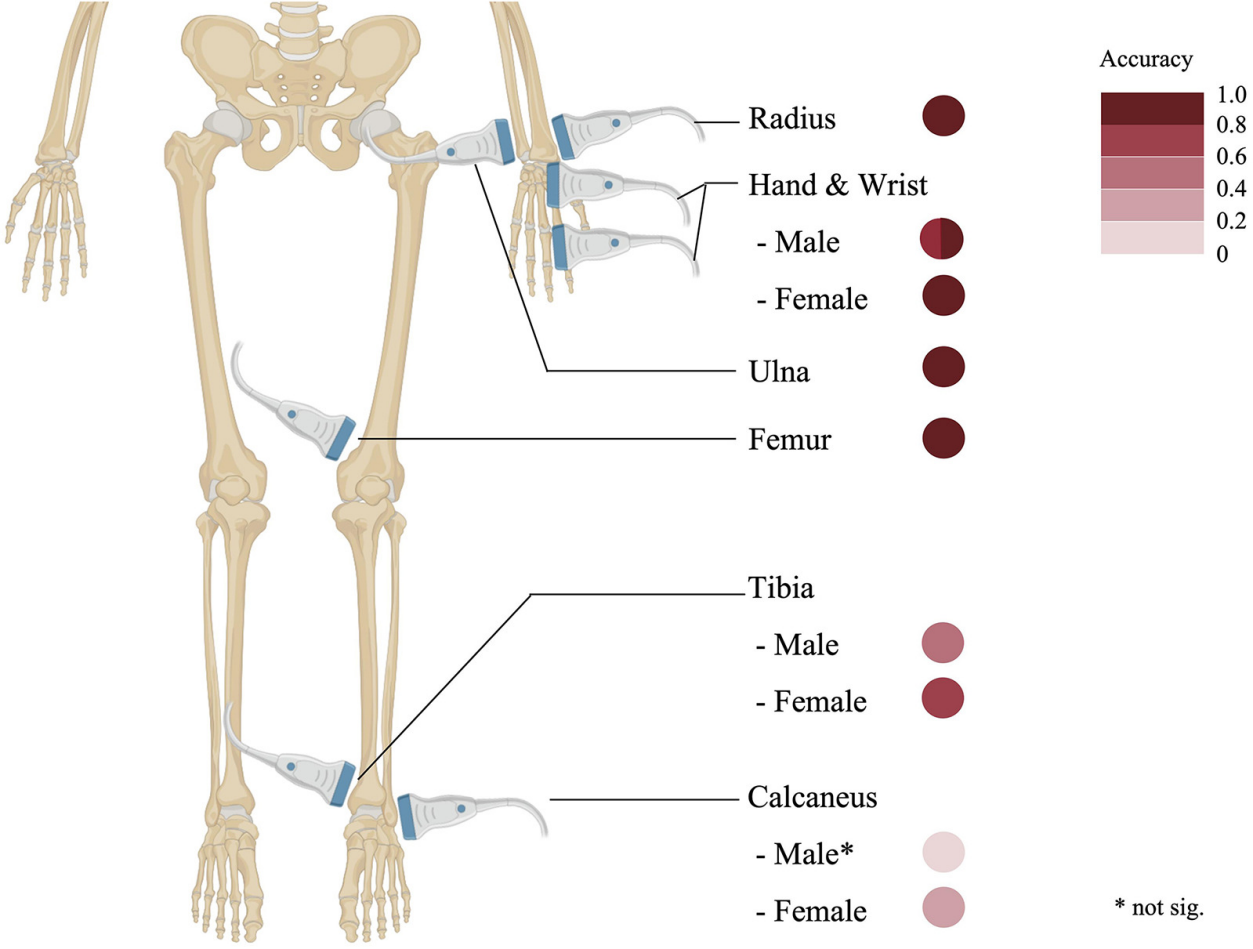
Commonly scanned skeletal sites and their accuracy.

Unlike radiographic methods that predominantly focus on the phalanges, the distal radius and ulna sites provide equally accessible epiphyseal visualization while enabling a larger probe positioning and a clearer display. Nonetheless, discrepancies have been observed between conventional radiographic and ultrasonographic measurements of the radial epiphysis (9). The wrist and phalanges also serve as common targets for ultrasonographic bone age assessment, delivering detailed epiphyseal-diaphyseal visualizations comparable to those in radiographic studies (9, 22). Additionally, sites such as the femur and medial epicondyle have been successfully utilized in ultrasound-based evaluations. Notably, the femur's ossification ratio exhibits a robust correlation with radiographic bone age, affirming its reliability as a parameter for assessing skeletal maturity (12). A detailed depiction of these frequently scanned skeletal regions and their accuracy parameters (compared to radiologic bone age assessment) is provided in Figure 2.

The hand-wrist complex demonstrates the highest correlation (*r* = 0.745–0.994 in males, 0.847–0.986 in females), followed by long bones (radius: *r* = 0.87; ulna: *r* = 0.89; femur: *r* = 0.90). The foot sites show greater variability, with tibial correlations of 0.50 (male) and 0.76 (female), while calcaneal measurements exhibit weaker associations [no significant relation [male] and 0.38 [female]]. Data derived from (8, 9, 13, 15, 20).

Performing multi-site assessments in ultrasound bone age evaluation enhances the reliability and accuracy of skeletal maturity assessments. Multi-site assessments, involving the evaluation of multiple skeletal sites, provide a more comprehensive picture of overall skeletal maturity. Studies have shown that the sum of ossification ratios of the radius, ulna, and femur provides a strong correlation with radiographic bone age (12). Multi-site assessments improve diagnostic accuracy by considering the variability in maturation rates among different skeletal regions. Some ossification centers may mature earlier or later than others, and evaluating multiple sites can help to identify any discrepancies and provide a more accurate overall assessment of skeletal maturity. Combining measurements from different sites, such as the radius, ulna, and femur, has been shown to improve the correlation with radiographic bone age compared to using a single site alone (13).

Most studies on bone age assessment utilize unilateral sites; however, some research incorporates bilateral sites for comparative analysis. For instance, one 100-sample study evaluated bilateral wrist joints using the BonAge system and found strong consistency between the left and right sides (*R*^2^ = 94.1, *P* < 0.001) (22). Another study (*n* = 192) revealed that the ossification ratios on girls' right side (dominant hand) were significantly higher than those on the left, suggesting a right-sided bone development advantage. In boys, no statistically significant difference in bone age between the left and right sides was observed. Notably, the discrepancy in ultrasound bone age between the left and right for both male and female children is relatively small, averaging approximately ± 0.6–0.8 years (23). These findings collectively suggest that unilateral evaluations show good reliability, though the incorporation of bilateral assessments may provide additional insights into skeletal maturity.

## 4 Comparison between ultrasound and traditional radiographic methods

### 4.1 Correlations

A number of studies have directly compared ultrasound-based bone age estimation with conventional radiographic methods.

Bilgili et al. (8) demonstrated that the ultrasonographic version of the GP atlas yielded significant correlations between bone ages determined via conventional radiographs and ultrasound examinations in 97 children (ranging in age from birth to 6 years), with 71.1% of males and 65.5% of females showing identical assessments and most differences being less than 6 months. However, although this study used blinding, both ultrasound and x-ray were evaluated by only one radiologist without consistency checks, which reduced the reliability of its results.

Ağırman et al. (9) assessed pubertal growth and bone age in 120 children aged 10–17 years using both imaging modalities (5-stage scoring system and GP atlas), finding no statistically significant differences in most measurements, thus confirming the overall compatibility of ultrasound-based assessments with conventional radiography (*r* = 0.745 for male, 0.847 for female).

One prospective study recruited a cohort of 1,089 Chinese children, ranging from newborns to 19 years old, and split them into a normal group (929) and a validation group (160) to assess the ossification ratios and SMS of radius, ulna, and femur (13). This research demonstrated outstanding diagnostic performance in detecting abnormal bone age, achieving a sensitivity of 93% and specificity of 98%, thereby establishing ultrasound-derived ossification ratios as a reliable quantitative tool.

Another prospective study conducted by Lv et al. (20) introduced an ultrasonic SMS system based on ossification ratios of the radius, ulna, and femur. This system demonstrated high diagnostic accuracy in evaluating bone age, with sensitivity reaching 97% and specificity of 98% (*n* = 442).

Other investigations have focused on comparing quantitative ultrasound techniques. For example, a study on a Dutch Caucasian pediatric population revealed that calcaneal and tibial quantitative ultrasound measurements had modest yet significant correlations with skeletal age—particularly in girls where parameters such as speed of sound (SOS) and broadband ultrasound attenuation (BUA) were positively related to bone maturation (15).

Furthermore, the assessment of skeletal age at the wrist using a novel ultrasound device, specifically the BonAge system, has demonstrated high agreement with the GP method, with correlation coefficients around 0.82 and mean absolute differences comparable to traditional radiographic evaluations (17).

### 4.2 Limitations and systematic biases

Some studies reveal a significant correlation between ultrasound and radiography, while others report inconsistencies.

For example, Widmer et al. (24) noted an intraclass correlation coefficient of merely 0.48 when comparing ultrasound-derived bone age from SMS with chronological age. However, the study's limited sample size of 24 males may undermine the reliability of these results, especially considering that chronological age often fails to accurately represent bone development in small cohorts.

In a larger study comprising 120 samples, Lequin et al. (15) found no significant correlation in males and only a weak positive correlation in females between radiographic bone age and SOS (*r* = 0.38, *p* < 0.01). Since this study focused on the foot, the intricate bone structure and variable flexion and extension in the area likely influenced the assessment of penetration time and speed. The variability in ultrasound results could also be influenced by operator skill and experience, which can introduce inconsistencies. Additionally, observer bias raises concerns, as systematic biases in ultrasound assessments often stem from imaging artifacts (9).

Additional research underscores the discrepancies between ultrasound and traditional radiographic bone age assessment, especially in cases categorized as delayed or advanced (22). In this blinded study involving 100 children, ultrasound was found to overestimate delayed bone age while underestimating advanced bone age. In younger individuals, the incomplete ossification process can result in poorly defined ossification centers, leading to an overestimation of bone age through ultrasound. Conversely, in older individuals, the nearly complete fusion of ossification centers diminishes the visibility of fusion lines, which can result in an underestimation of bone age (22). These factors underscore the need for cautious interpretation of ultrasound bone age assessments and suggest that further studies are necessary to enhance the method's reliability.

The primary research findings about the correlations and limitations are presented in Table 1.

**Table 1:** Correlations and limitations of ultrasound bone age assessment vs. traditional radiographic method.

Study (Year)	Population (Sample Size, Age)	Measurement Site	Ultrasound Technique	Reference Standard	Reliability	Accuracy (Correlation coefficients)
Lequin et al. (15)	120 (53 males), 7–19 years	Calcaneus and tibia	Ultrasound densitometer, QUS (Quantitative Ultrasound): SOS (Speed of Sound), BUA (Broadband Ultrasound Attenuation), QUI (Quantitative Ultrasound Index)	RBA (Radiographic Bone Age) (GP atlas, Greulich-Pyle atlas)	CV = 0.2% (SOS), 3.5% (BUA), 1.9% (QUI)	Calcaneus: 0.26 (not sig., male), 0.38 (female) Tibia: 0.50 (male) 0.76 (female)
Bilgili et al. (8)	97 (45 males), 0–6 years	Left hand and wrist	B-mode ultrasound, ultrasonographic version of the GP atlas	RBA (GP atlas)	Not mentioned	0.994 (male), 0.986 (female)
Mentzel et al. (17)	70 (36 males), 6–17 years	Distal radial and ulna	The BonAge system, SOS	RBA (GP atlas)	Not mentioned	0.82
Khan et al. (22)	100 (50 males), 2.3–19.4 years	Bilateral wrists	The BonAge system, SOS	RBA (GP atlas and TW3, Tanner and Whitehouse 3)	Not mentioned	74.6–82.6 (r^2^)
Utczas et al. (26)	1,502 (760 males), 6–18 years	Left hand and wrist	The BonAge system, SOS	RBA (GP atlas)	Not mentioned	0.895–0.958
Ağırman et al. (9)	120 (82 females), 10–17 years	Phalanges, sesamoid bones, radial epiphysis	B-mode ultrasound, 5-stage scoring system and GP atlas	RBA and CA (GP atlas)	ICC: 0.96	CA (Chronological Age): 0.891(male), 0.780(female) RBA: 0.745(male), 0.847(female)
Wan et al. (13)	1,089 (578 males), 3–14 years (interquartile range)	Radius, ulna, and femur	B-mode ultrasound, SMS (Skeletal Maturity score) and Box-Cox power exponential (BCPE) distribution	RBA (TW3)	ICC: 0.97	Sensitivity: 93%(male), 100% (female) Specificity: 98% (male), 98% (female)
Lv et al. (20)	442 (257 females), 1–18 years	Radius, ulna, and femur	B-mode ultrasound; SMS and the formulas fitting TW3	RBA (TW3)	ICC: 0.993	Sensitivity: 97% Specificity: 98%

### 4.3 Radiation safety, time-consuming and accessibility

One of the most significant advantages of ultrasound over traditional radiographic methods is that it eliminates exposure to ionizing radiation. While the radiation dose from a single bone age x-ray is extremely low (<0.00012 mSv) (3), pediatric patients remain particularly susceptible to the harmful effects of radiation. This susceptibility underscores the importance of adhering to the ALARA principle (As Low As Reasonably Achievable), which advocates for minimizing radiation exposure, especially in vulnerable populations such as children (5). The need to reduce radiation becomes even more critical in conditions that require regular imaging for monitoring, such as growth hormone deficiency, precocious puberty, and scoliosis (22, 25). By offering a radiation-free alternative, ultrasound emerges as a safer and more sustainable diagnostic tool for these pediatric cases.

The examination time for both methods—x-ray and ultrasound—is relatively short. X-ray inspections typically take only a few seconds to complete. In contrast, the time required for ultrasound-based examinations can vary depending on the specific method employed and the operator's level of experience. For instance, during ultrasound examinations of the left hand and knee, less experienced operators reported an average examination time of 3 min (±2 min), whereas more experienced operators reduced this time to just 1 min (±1 min) (13). When using the ultrasonographic GP Atlas, the average time to evaluate each patient during the first three weeks of a study was measured at 3 min and 45 s (±12 s). However, as operators gained experience, the average examination time decreased to 2 min and 12 s (±10 s) per patient (8). In the context of BAUSport ultrasound examinations, the total time required to complete the assessment is typically longer, ranging from approximately 5–10 min per participant (21).

Although radiography is widely used in clinical practice, it remains dependent on fixed infrastructure and radiation shielding, limiting its accessibility in resource-constrained settings. Ultrasound devices are portable and free from ionizing radiation, which can be utilized in low-resource settings where traditional radiography may be impractical. Ultrasound's accessibility makes ultrasound a viable option for large-scale screening programs (26). However, operator dependency and variability in image interpretation remain challenges for ultrasound, whereas radiography provides standardized, high-resolution images.

Table 2 provides a side-by-side comparison of radiographic and ultrasound bone age assessment performance. As illustrated, each technique possesses unique strengths and limitations, highlighting the importance of selecting the method based on specific clinical and operational requirements.

**Table 2:** Comparative table showing radiographic vs. ultrasound bone age assessment performance side-by-side.

Parameters	Radiographic bone age assessment	Ultrasound bone age assessment
Sensitivity and Specificity	1.00 (reference)	93–98%^a^ and 98–100%^a^
Bias	Standardized images	Systematic bias
Time per exam	Several seconds	1–10 min^b^
Radiation-free	No	Yes
Follow-up Frequency	Minimum safe interval	Anytime

^a^ Representative sensitivity/specificity values from Wan et al. (13) and Lv et al. (5).

^b^ Time estimates derived from Wan et al. (13), Bilgili et al. (8), and Cumming et al. (21).

## 5 Challenges and future directions

### 5.1 Current technical and operational limitations

Ultrasound-based bone age assessment, despite its advantages, still faces several technical and operational limitations that hinder its widespread adoption and use as a standard method for bone age evaluation in adolescents. These limitations impact the efficacy and reliability of ultrasound, posing challenges in clinical settings and epidemiological studies.

Operator skill and experience significantly influence the outcomes of ultrasound-based bone age assessments. The acquisition and interpretation of ultrasound images require a high level of expertise to ensure accuracy and consistency. The quality of the ultrasound image and the precision of measurements are highly dependent on the operator's technique, including probe positioning, image optimization, and anatomical knowledge (9).

Variability in operator skills can lead to subjective variances in assessments and affect inter-operator reliability. To mitigate this, targeted training programs for healthcare professionals are essential. These programs should focus on standardized scanning protocols, anatomical landmarks, and image interpretation criteria. A study has found that inexperienced inspectors can also obtain reliable bone maturity data through standardized training (27). Establishing a unified training curriculum can increase the accessibility of bone age ultrasound assessment and ensure the stability of bone age data. Also, the development of user-friendly software with automated guidance and quality control features can further reduce operator dependency and improve the reliability of ultrasound assessments.

### 5.2 Standardization and population-specific adaptations

Standardization in ultrasound methodologies is crucial for bone age assessment to ensure clinical reliability and comparability across studies. Consistency in protocols is essential because the lack of standardized procedures complicates comparisons across different research findings and clinical practices. Without uniformity, variations in scanning techniques, measurement parameters, and interpretation criteria can lead to discrepancies and limit the applicability of ultrasound in diverse settings (9).

#### 5.2.1 Need for consensus on protocols and measurement criteria

Reaching a consensus among professionals regarding ultrasound protocols and measurement criteria is of paramount importance. Current disparities in practices include variations in the skeletal sites scanned, the ultrasound parameters measured, and the methods used to calculate bone age. For instance, some studies focus on the distal radius and ulna (13), while others evaluate the femur and tibia. Similarly, different studies may use ossification ratios or other acoustic measures, such as speed of sound (SOS) and broadband ultrasound attenuation (BUA) (15).

To achieve consensus, a collaborative effort involving researchers, clinicians, and regulatory bodies is needed. Potential frameworks for achieving consensus include:

1. Expert Panels: Establishing expert panels to develop and disseminate standardized protocols and measurement criteria. These panels should include representatives from various disciplines, such as radiology, endocrinology, orthopedics, and ultrasound technology.
2. Consensus Conferences: Organizing consensus conferences to discuss and resolve discrepancies in current practices. These conferences can provide a platform for sharing knowledge, exchanging ideas, and reaching agreement on best practices.
3. Guidelines and Recommendations: Developing clinical guidelines and recommendations based on the best available evidence. These guidelines should outline the recommended scanning protocols, measurement parameters, and interpretation criteria for ultrasound-based bone age assessment.
4. Training and Education: Providing training and education programs to ensure that examiners are proficient in the standardized protocols and measurement criteria.
5. Large-scale longitudinal validation: Implementing the multi-center alliance framework through the integration of standardized collection protocols and validated software tools for automated quality control.

#### 5.2.2 Development of comprehensive, multi-ethnic reference databases

Establishing comprehensive, multi-ethnic reference databases is essential for improving the accuracy of bone age assessments in diverse populations. Bone age can vary significantly across different ethnic groups due to genetic, environmental, and nutritional factors (4, 28). The use of reference data derived from one population may lead to inaccurate assessments and inappropriate clinical decisions when applied to individuals from different ethnic backgrounds.

Multi-ethnic reference databases can improve the accuracy of bone age assessments in various demographic groups and facilitate proper clinical decision-making (29). These databases should include data on a wide range of ethnicity, ages, and genders, allowing for the development of population-specific reference ranges. The collection of data should be standardized to ensure consistency and comparability across different populations.

### 5.3 Integration with artificial intelligence and machine learning

Artificial intelligence (AI) and machine learning have the potential to transform ultrasound-based assessments. AI algorithms can be trained to automatically analyze ultrasound images, identify key anatomical landmarks, and measure ossification ratios with remarkable precision. This automation can significantly reduce operator dependence and enhance the reliability of bone age assessments (30). An automated algorithm, BAUSport, has been employed to estimate skeletal age in young athletes (21). This innovation not only minimizes the time and effort required for manual measurements but also improves the accuracy of the results.

Potential applications of AI and machine learning in ultrasound-based bone age assessment include:

1. Predictive Algorithms: Machine learning models can be trained to predict bone age based on a combination of ultrasound parameters and clinical data. These models can provide clinicians with valuable insights into the skeletal maturity of adolescents and help guide treatment decisions.
2. Quality Control: AI can be used to assess the quality of ultrasound images and identify potential errors or biases. This can help ensure that the images are of sufficient quality for accurate bone age assessment.
3. Personalized Assessments: AI can enhance bone age assessments by integrating individual factors such as age, gender, genetic background, and socio-environmental influences (e.g., nutrition and obesity rates). By accounting for both biological and lifestyle-related variations, AI models can improve assessment accuracy and support personalized clinical decisions.

The implications of AI and machine learning for improving diagnostic accuracy and efficiency are substantial. Automated image analysis can reduce the time required for bone age assessment, allowing clinicians to focus on other aspects of patient care. Predictive algorithms can provide valuable insights into the skeletal maturity of adolescents, helping guide treatment decisions and improve clinical outcomes.

## 6 Conclusion

Ultrasound-based bone age assessment offers a promising, radiation-free, and non-invasive alternative to traditional radiographic methods, particularly well-suited for pediatric patients. Studies have demonstrated that ultrasound bone age assessment correlates well with established radiographic standards, highlighting its potential accuracy and reliability. Key limitations include the lack of standardized scanning protocols, variability in results across different populations, declining sensitivity in younger or older children, and the technique's dependence on operator skill and expertise. Additionally, unlike radiographic assessments, ultrasound outcomes can be significantly influenced by the choice of bone site and technical proficiency. Another critical gap is the absence of large-scale, longitudinal validation studies to confirm its long-term accuracy and clinical utility across diverse pediatric populations. To advance the clinical application of ultrasound-based bone age assessment, future research should prioritize the development of standardized protocols, the creation of large-scale, longitudinal and multi-ethnic reference databases, and the integration of artificial intelligence to improve precision and reduce subjectivity. Addressing these challenges will be essential for optimizing the reliability and utility of ultrasound bone age assessment, ultimately enabling it to become a robust tool in pediatric clinical practice.

## References

[1] Xie M Chagin AS. The epiphyseal secondary ossification center: evolution, development and function. Bone. (2020) 142:115701. 10.1016/j.bone.2020.115701 33091640

[2] Gilsanz V Chalfant J Kalkwarf H Zemel B Lappe J Oberfield S Age at onset of puberty predicts bone mass in young adulthood. J Pediatr. (2010) 158(1):100–5; 105.e1–2. 10.1016/j.jpeds.2010.06.054 20797727 PMC4767165

[3] Cavallo F Mohn A Chiarelli F Giannini C. Evaluation of bone age in children: a mini-review. Front Pediatr. (2021) 9:580314. 10.3389/fped.2021.580314 33777857 PMC7994346

[4] Mughal AM Hassan N Ahmed A. Bone age assessment methods: a critical review. Pak J Med Sci. (2014) 30(1):211–5. 10.12669/pjms.301.4295 24639863 PMC3955574

[5] Ekizoglu O Er A Buyuktoka AD Bozdag M Karaman G Moghaddam N Ultrasonographic assessment of ossification of the distal radial epiphysis for estimating forensic age.international. J Legal Med. (2021) 135(4):1573–80. 10.1007/s00414-021-02521-2 33611667 PMC8206057

[6] Wagner UA Diedrich V Schmitt O. Determination of skeletal maturity by ultrasound: a preliminary report. Skeletal Radiol. (1995) 24(6):417–20. 10.1007/bf00941236 7481897

[7] Jensen JA. Medical ultrasound imaging. Prog Biophys Mol Biol. (2006) 93(1–3):153–65. 10.1016/j.pbiomolbio.2006.07.025 17092547

[8] Bilgili Y Hizel S Kara SA Sanli C Erdal HH Altinok D. Accuracy of skeletal age assessment in children from birth to 6 years of age with the ultrasonographic version of the greulich-pyle atlas. J Ultrasound Med. (2003) 22(7):683–90. 10.7863/jum.2003.22.7.683 12862266

[9] Ağırman KT Bilge OM Miloğlu Ö. Ultrasonography in determining pubertal growth and bone age. Dentomaxillofac Radiol. (2018) 47(7):20170398. 10.1259/dmfr.20170398 29668314 PMC6196061

[10] Rüeger E Hutmacher N Eichelberger P Löcherbach C Albrecht S Romann M. Ultrasound imaging-based methods for assessing biological maturity during adolescence and possible application in youth sport: a scoping review. Children (Basel, Switzerland). (2022) 9(12):1985. 10.3390/children9121985 36553428 PMC9776568

[11] Schmidt S Schiborr M Pfeiffer H Schmeling A Schulz R. Age dependence of epiphyseal ossification of the distal radius in ultrasound diagnostics. Int J Leg Med. (2013) 127(4):831–8. 10.1007/s00414-013-0871-2 23708645

[12] Wan J Zhao Y Feng Q Zhang C. Summation of ossification ratios of radius, Ulna and Femur: a new parameter to evaluate bone age by ultrasound. Ultrasound Med Biol. (2020) 46(7):1761–68. 10.1016/j.ultrasmedbio.2020.03.021 32402669

[13] Wan J Zhao Y Feng Q Lv P Hong K Zhang C. Statistical confirmation of a method of US determination of bone age. Radiology. (2021) 300(1):176–83. 10.1148/radiol.2021204353 34003051

[14] Qiao Y Lv P Hong K Zhao Y Feng Q Zhang C. Use of the ultrasound bone maturity indexes to assess whether children have reached their final height. Ultrasound Med Biol. (2025) 51(5):903–8. 10.1016/j.ultrasmedbio.2025.02.004 39952823

[15] Lequin MH Hop WCJ van Rijn RR Bukkems MCHW Verhaak LLJ Robben SGF Comparison between quantitative calcaneal and tibial ultrasound in a Dutch Caucasian pediatric and adolescent population. J Clin Densitom. (2001) 4(2):137–46. 10.1385/jcd:4:2:137 11477307

[16] Pettinato AA Loud KJ Bristol SK Feldman HA Gordon CM. Effects of nutrition, puberty, and gender on bone ultrasound measurements in adolescents and young adults. J Adolesc Health. (2006) 39(6):828–34. 10.1016/j.jadohealth.2006.04.015 17116512

[17] Mentzel HJ Vilser C Eulenstein M Schwartz T Vogt S Böttcher J Assessment of skeletal age at the wrist in children with a new ultrasound device. Pediatr Radiol. (2005) 35(4):429–33. 10.1007/s00247-004-1385-3 15729586

[18] Ramírez-Vélez R Ojeda-Pardo ML Correa-Bautista JE González-Ruíz K Navarro-Pérez CF González-Jiménez E Normative data for calcaneal broadband ultrasound attenuation among children and adolescents from Colombia: the FUPRECOL study. Arch Osteoporos. (2015) 11:2. 10.1007/s11657-015-0253-0 26691632

[19] Travers-Gustafson D Stegman MR Heaney RP Recker RR. Ultrasound, densitometry, and extraskeletal appendicular fracture risk factors: a cross-sectional report on the saunders county bone quality study. Calcif Tissue Int. (1995) 57(4):267–71. 10.1007/bf00298881 8673863

[20] Lv P Zhang C. Tanner-Whitehouse skeletal maturity score derived from ultrasound images to evaluate bone age. Eur Radiol. (2022) 33(4):2399–406. 10.1007/s00330-022-09285-2 36462047 PMC10017602

[21] Cumming SP Pi-rusiñol R Rodas G Drobnic F Rogol AD. The validity of automatic methods for estimating skeletal age in young athletes: a comparison of the BAUSport ultrasound system and BoneXpert with the radiographic method of fels. Biol Sport. (2023) 41(1):61–7. 10.5114/biolsport.2024.127380 38188108 PMC10765447

[22] Khan KM Miller BS Hoggard E Somani A Sarafoglou K. Application of ultrasound for bone age estimation in clinical practice. J Pediatr. (2008) 154(2):243–7. 10.1016/j.jpeds.2008.08.018 18823906

[23] Zhao Y Wan J Lv P Zhang C. Bone age evaluated with conventional ultrasound: the inter-side difference. J Ultrasound Med. (2023) 42(6):1249–56. 10.1002/jum.16136 36480130

[24] Widmer C Busch JD Born D Romann M. Assessment of biological age with conventional ultrasound imaging as an alternative to x-ray-A pilot study in youth soccer. Eur J Sport Sci. (2025) 25(3):e12264. 10.1002/ejsc.12264 39910800 PMC11799066

[25] Thaler M Kaufmann G Steingruber I Mayr E Liebensteiner M Bach C. Radiographic versus ultrasound evaluation of the risser grade in adolescent idiopathic scoliosis: a prospective study of 46 patients. Eur Spine J. (2008) 17(9):1251–5. 10.1007/s00586-008-0726-6 18663485 PMC2527420

[26] Utczas K Muzsnai A Cameron N Zsakai A Bodzsar EB. A comparison of skeletal maturity assessed by radiological and ultrasonic methods. Am J Hum Biol. (2017) 29(4):e22966. 10.1002/ajhb.22966 28094893

[27] Hutmacher N Busch JD Rüeger E Romann M Eichelberger P. Reliability of two recently developed procedures assessing biological maturity by ultrasound imaging-A pilot study. Children (Basel, Switzerland). (2024) 11(3):326. 10.3390/children11030326 38539360 PMC10968870

[28] Ontell FK Ivanovic M Ablin DS Barlow TW. Bone age in children of diverse ethnicity. Am J Roentgenol. (1996) 167(6):1395–8. 10.2214/ajr.167.6.8956565 8956565

[29] Novotny R Davis J. Growth in bone and body size among Asian and white girls in the female adolescent maturation (FAM) study. Arch Osteoporos. (2015) 10:31. 10.1007/s11657-015-0234-3 26373971

[30] Zadoo N Tak N Reddy AJ Patel R. Enhancing pediatric bone age assessment using artificial intelligence: implications for orthopedic surgery. Cureus. (2025) 17(2):e79507. 10.7759/cureus.79507 39989489 PMC11847569

